# A1298C polymorphism in the *MTHFR *gene predisposes to cardiovascular risk in rheumatoid arthritis

**DOI:** 10.1186/ar2989

**Published:** 2010-04-26

**Authors:** Rogelio Palomino-Morales, Carlos Gonzalez-Juanatey, Tomas R Vazquez-Rodriguez, Luis Rodriguez, Jose A Miranda-Filloy, Benjamin Fernandez-Gutierrez, Javier Llorca, Javier Martin, Miguel A Gonzalez-Gay

**Affiliations:** 1 Instituto de Parasitología y Biomedicina Lopez-Neyra, CSIC, Parque Tecnológico de Ciencias de la Salud, Avenida del Conocimiento s/n Armilla, Granada 18100, Spain; 2 Cardiology Division, Hospital Xeral-Calde, c/Dr. Ochoa, Lugo 27004, Spain; 3 Rheumatology Division, Hospital Xeral-Calde, c/Dr. Ochoa, Lugo 27004, Spain; 4 Rheumatology Service, Hospital Clínico San Carlos, c/Profesor Martín Lagos, S/N Madrid 28040, Spain; 5 Division of Epidemiology and Computational Biology, School of Medicine, University of Cantabria, and CIBER Epidemiología y Salud Pública (CIBERESP), Avda. Herrera Oria s/n, 39011 Santander, Spain; 6 Rheumatology Division, Hospital Universitario Marques de Valdecilla, Avenida de Valdecilla s/n, 39008, Santander, Spain

## Abstract

**Introduction:**

We determined the contribution of the methylene tetrahydrofolate reductase (*MTHFR*) 677 C>T and 1298 A>C gene polymorphisms to the susceptibility to rheumatoid arthritis (RA). We also assessed whether these two *MTHFR *gene polymorphisms may be implicated in the development of cardiovascular (CV) events and subclinical atherosclerosis manifested by the presence of endothelial dysfunction, in a series of Spanish patients with RA.

**Methods:**

Six hundred and twelve patients fulfilling the 1987 American College of Rheumatology classification criteria for RA, seen at the rheumatology outpatient clinics of Hospital Xeral-Calde, Lugo and Hospital San Carlos, Madrid, were studied. Patients and controls (n = 865) were genotyped using predesigned TaqMan SNP genotyping assays.

**Results:**

No significant differences in allele or genotype frequencies for the *MTHFR *gene polymorphisms between RA patients and controls were found. Also, no association between the *MTHFR *677 C>T polymorphism and CV events or endothelial dysfunction was observed. However, the *MTHFR *1298 allele C frequency was increased in patients with CV events after 5 years (38.7% versus 30.3%; odds ratio = 1.45; 95% confidence interval = 1.00 to 2.10; *P *= 0.04) and 10 years (42.2% versus 31.0%; odds ratio = 1.62; 95% confidence interval = 1.08 to 2.43; *P *= 0.01) follow up. Moreover, patients carrying the *MTHFR *1298 AC and CC genotypes had a significantly decreased flow-mediated endothelium-dependent vasodilatation (4.3 ± 3.9%) compared with those carrying the *MTHFR *1298 AA genotype (6.5 ± 4.4%) (*P *= 0.005).

**Conclusions:**

Our results show that the *MTHFR *1298 A>C gene polymorphism confers an increased risk for subclinical atherosclerosis and CV events in patients with RA.

## Introduction

Patients with rheumatoid arthritis (RA) have increased risk of cardiovascular (CV) disease due to accelerated atherosclerosis [1]. Besides classic CV risk factors, a number of nontraditional CV risk factors have also been implicated in the elevated CV mortality observed in these patients [2].

In this regard, chronic inflammation and the genetic background increase the risk of CV events in RA regardless of the presence of traditional CV risk factors [3]. Hyperhomocysteinemia has been found to be an independent nontraditional risk factor for CV disease, including coronary disease, in the general population [4]. Homocysteine is an intermediary amino acid formed during the conversion of methionine to cysteine. High elevations may be seen in uncommon autosomal defects of the metabolizing enzymes cystathionine β-synthase and 5,10-methylene tetrahydrofolate reductase (MTHFR) [5]. Less severe elevations of homocysteine levels are more commonly observed as a result of heterozygous mutations of these enzymes, dietary deficits of folate or vitamin B_12_, or in patients with liver disease or decreased renal function [6]. Homocysteine is directly toxic to endothelial cells, increases low-density lipoprotein oxidation, and has prothrombotic effects [7].

Increased levels of homocysteine have been found in patients with RA [7]. Although significant survival benefit, largely by reducing CV mortality, has been also observed following methotrexate (MTX) therapy [8], this medication reduces levels of plasma and red blood cell folate, which increases homocysteine levels via reduced activity of MTHFR [5]. Some investigators have thus advocated supplementation with folic acid for long-term, low-dose MTX therapy, since folic acid supplementation prevents MTX toxicity and hyperhomocysteinemia [9].

A common C677T polymorphism in the gene coding for the MTHFR enzyme has been found to be a new candidate genetic risk factor for CV disease in the general population [10]. This mutation determines a temperature-related loss of function, with the T allele having an enzyme activity of approximately 35% of the values observed in individuals carrying the C allele. The 677TT genotype is associated with significantly higher total plasma homocysteine levels than in heterozygotes or in individuals with wild-type C alleles [10].

The A1298C polymorphism in the *MTHFR *gene has also been associated with MTHFR activity [11,12]. This A1298C polymorphism is known to have a lower effect in reducing enzyme activity, compared with the 677 mutation. This is more pronounced in the homozygous (CC) state than in the heterozygous (AC) or normal (AA) states. Heterozygote individuals for both the C677T and the A1298C mutations were found to exhibit 50 to 60% of control activity, a value lower than that seen in single heterozygotes for the C677T variant [11]. Interestingly, a recent study has disclosed an association of the A1298C polymorphism in the *MTHFR *gene with susceptibility to RA in Southern European individuals [13].

Taking all these considerations together, in the present study we assessed the potential contribution of the *MTHFR *677 C>T and 1298 A>C gene polymorphisms to disease susceptibility of patients with RA. In a further step we aimed to determine whether these two functional *MTHFR *gene polymorphisms might be associated with the increased incidence of CV events observed in patients with RA. Moreover, we assessed whether these two *MTHFR *gene polymorphisms might be associated with an increased risk of and subclinical atherosclerosis manifested by the presence of endothelial dysfunction in RA.

## Materials and methods

### Patients and controls

Six-hundred and twelve consecutive patients who fulfilled the 1987 American College of Rheumatology classification criteria for RA [14], seen at the rheumatology outpatient clinics of Hospital Xeral-Calde, Lugo and Hospital Clínico San Carlos, Madrid between March 1996 and January 2006, and 865 controls, matched by age, sex and ethnicity, from the same regions were assessed for differences in the *MTHFR *gene biallelic polymorphisms C677T and A1298C. Information on the main demographic characteristics of this Caucasian cohort is presented in Table 1.

**Table 1:** Demographic characteristics and genotype distribution of rheumatoid arthritis patients included in the study

Patients (*n*)	612
Main characteristics	
Age at disease onset (years)	49.0 ± 15.1
Follow up (years)	14.3 ± 9.4
Women (%)	74.5
Rheumatoid factor positive (%)	68.5
Anti-CCP antibodies positive (%)	66.9
Shared epitope positive (%)	63.6
Extra-articular manifestations (%)	21.2
Radiographic erosions in hands and/or feet (%)	64.9
*MTHFR *677 C>T polymorphism	
CC	257 (42.9)
CT	275 (45.9)
TT	67 (11.2)
*MTHFR *1298 A>C polymorphism	
AA	286 (48.6)
AC	242 (41.2)
CC	60 (10.2)
Medication (%)	
Patients receiving DMARDs	90.1
Patients on treatment with methotrexate	84.6
Patients on treatment with TNFα blockers	17.1
Cardiovascular risk factors (%)	
Hypercholesterolemia and/or hypertriglyceridemia	19.2
Hypertension	16.1
Diabetes mellitus	2.8
Obesity	5.4
Smoking	9.1
Patients with cardiovascular events^a^	80 (13.1)
Ischemic heart disease	37 (4.6)
Heart failure	5 (2.3)
Cerebrovascular accidents	32 (5.1)
Peripheral arteriopathy	6 (1.0)

Data presented as mean ± standard deviation or *n*(%), except where indicated otherwise. CCP, cyclic citrullinated peptide; DMARDs, disease-modifying anti-rheumatic drugs. ^a^This category was defined considering the first type of cardiovascular event.

A CV event was considered to be present if the patient had ischemic heart disease, heart failure, cerebrovascular accident or peripheral arteriopathy. Eighty (13.1%) out of the 612 patients with RA experienced CV events.

### Study protocol

At the time of recruitment, patients' data regarding traditional CV risk factors, previous history of CV events, and clinical manifestations were registered. Patients were followed and assessed every 3 to 6 months and patients' medical records were screened for comorbidities. Clinical definitions were established as previously described [3,15,16]. Patients were prospectively followed and clinical records were examined until patient death or to 1 January 2008.

Since Hospital Xeral-Calde and Hospital Clínico San Carlos are the referral centers for the population of each respective area, the first CV event was defined as an event (case) of CV complication diagnosed at the hospital in a patient without a previous history of CV disease. Specific information on CV events was collected based on patients' medical records.

Based on previously established protocols of management, all patients on MTX therapy were treated with folate supplementation. With respect to this, MTX-treated patients from Hospital Xeral-Calde received folic acid 7.5 mg/week. MTX-treated patients from Hospital Clínico were taking folic acid 10 mg/week.

Informed consent was obtained from all patients. The local institutional committees approved the study.

To determine the potential association between the *MTHFR *gene polymorphisms and the presence of subclinical atherosclerosis, between March and December 2007 a randomly selected subgroup of patients from Lugo was assessed for the presence of endothelial dysfunction by brachial artery reactivity study. For the purpose of assessment of endothelial dysfunction, however, patients with a history of CV events were excluded.

Endothelium-dependent, flow-mediated (post-ischemia) vasodilatation (FMD) and endothelium-independent (post-nitroglycerin) vasodilatation (NGT) were measured in 108 patients with RA from this series by brachial ultrasonography as previously reported [17-19]. Since we have observed a rapid but transient effect of the anti-TNFα monoclonal antibody infliximab that lasted 4 hours after the infusion of this drug in patients with RA [18], the assessment of the endothelial function was performed in 19 of the 108 patients undergoing TNFα blocker therapy (14 of them with infliximab, four with adalimumab and one with etanercept) 24 to 48 hours before the administration of the anti-TNFα blocker. Normal values for the FMD percentage vary from one laboratory to another: in the echocardiography laboratory of our center, adults with FMD percentage values < 7% are considered to have endothelial dysfunction [20]. Based on 32 controls, the intra-observer variability showed the following coefficients of variation: FMD, 1.3%; NTG, 1.9%.

### Genotyping

DNA from patients and controls was obtained from peripheral blood, using standard methods. Patients and controls were genotyped for the *MTHFR *677 C>T and 1298 A>C gene polymorphisms using a PCR system with a predeveloped TaqMan allelic discrimination assay (Applied Biosystems, Foster City, CA, USA). Allele-specific probes were labeled with the fluorescent dyes VIC and FAM, respectively. PCR was carried out in a total reaction volume of 4 μl with the following amplification protocol: denaturation at 95°C for 10 minutes, followed by 40 cycles of denaturation at 92°C for 15 seconds, finished with annealing and extension at 60°C for 1 minute. Post PCR, the genotype of each sample was attributed automatically by measuring the allele-specific fluorescence on the ABI PRIM 7900 Sequence Detection System using SDS 2.3 software for allelic discrimination (Applied Biosystems). Duplicate samples and negative controls were included to ensure accuracy of genotyping.

### Statistical analysis

Strength of association between RA and alleles or genotypes of the *MTHFR *gene polymorphism was estimated using odds ratios (ORs) and 95% confidence intervals (CIs). Levels of significance were determined using contingency tables by chi-square analysis.

The strength of association between CV events in RA and alleles or genotypes of polymorphisms in the *MTHFR *gene was estimated using the OR and 95% CI, via multiple logistic regression; estimates were adjusted by age at diagnosis of the disease (continuous), gender, age at the time of study (continuous), rheumatoid factor and traditional (classic) CV risk factors (presence/absence) as potential confounders.

The association between genotypes of the *MTHFR *gene polymorphisms and percentages of FMD and NTG was tested using one-way analysis of variance, and the unpaired t test was used to compare variables between two groups. Moreover, we also tested association between these parameters and genotypes using analysis of covariance, adjusting by gender, age and duration of the disease at the time of the ultrasonographic study (continuous), rheumatoid factor and traditional (classic) CV risk factors (presence/absence). Statistical significance was defined as P ≤ 0.05. Calculations were performed with the statistical package SPSS 15.0 for Windows (SPSS Inc., Chicago, IL, USA).

## Results

### Allele and genotype frequencies of the *MTFHR *polymorphisms in RA patients and controls

The study had 80% power to detect an OR of 1.7 and 94% power to detect an OR of 2.0. Genotype frequencies of the *MTHFR *variants studied were in Hardy-Weinberg equilibrium in patients and controls. The *MTHFR *677 C>T gene polymorphism in RA patients and controls did not show significant differences in the genotypic frequencies (RA patients: CC, 42.9%; CT, 45.9%; TT, 11.2%; and controls: CC, 42.9%; CT, 39.9%; TT, 17.2%) and the allelic frequencies (T-allele frequency, 34.1% and 37.2% in RA patients and controls, respectively). Similarly, the *MTHFR *1298 A>C assessment did not disclose significant differences in the genotypic and allelic frequencies between patients and controls (RA patients: AA, 48.6%; AC, 41.2%; CC, 10.2%; C-allele frequency, 30.8%; and controls: AA, 49.7%; AC, 40.2%; CC 10.1%; C-allele frequency, 30.2%). Also, no significant differences in the age at onset of the disease, rheumatoid factor, anti-cyclic citrullinated peptide antibodies, shared epitope, and age at the time of disease diagnosis were observed according to the different *MTHFR *genotypes in the series of RA patients (data not shown).

In addition, these two gene polymorphisms did not form haplotypes, with *R*^2 ^= 0.15. This result is similar to the data found in the hapmap database in a Caucasian population that show *R*^2 ^= 0.17 between both variants [21].

### *MTHFR *gene polymorphisms and cardiovascular events

Tables 2 and 3 present the genotype frequencies of the *MTHFR *gene polymorphisms assessed in this cohort of RA patients stratified by the presence of CV events. Interestingly, RA patients carrying the C allele of the *MTHFR *1298 A>C gene polymorphism (patients with *MTHFR *1298CC and *MTHFR *1298AC genotypes) presented a statistically significant increased risk of suffering CV events compared with those homozygous for *MTHFR *1298AA (*MTHFR *1298CC + *MTHFR *1928AC genotype frequency, 62.3% in RA patients with CV events versus 49.7% in those RA patients without CV events; OR = 1.67; 95% CI = 1.00 to 2.82; *P *= 0.04). This association was in keeping with a statistically significant increase of the C-allele frequency in patients with CV events (39.0%) compared with that observed in RA patients without CV events (29.5%) (OR = 1.52; 95% CI = 1.06 to 2.19; *P *= 0.02).

**Table 2:** Frequencies of gene polymorphisms with or without cardiovascular events: 5 years follow up

Polymorphism	Cardiovascular event occurrence in RA patients					
	
	Overall			5 years follow up		
		
	+	-	*P *value	+	-	*P *value
*MTHFR *1298 A>C	(n = 77)	(n = 511)		(n = 75)	(n = 484)	
AA	29 (37.7)	257 (50.3)	Ref.	29 (38.7)	241 (49.8)	Ref.
AC	36 (46.8)	206 (40.3)	0.10	34 (45.3)	193 (39.9)	0.16
CC	12 (15.6)	48 (9.4)	0.03	12 (16.0)	50 (10.3)	0.04
A	94 (61.0)	720 (70.5)	Ref.	92 (61.3)	675 (69.7)	Ref.
C	60 (39.0)	302 (29.5)	0.02	58 (38.7)	293 (30.3)	0.04
*MTHFR *677 C>T	(n = 76)	(n = 523)		(n = 75)	(n = 492)	
CC	38 (50.0)	219 (41.9)	Ref.	38 (50.7)	210 (42.7)	Ref.
CT	28 (36.8)	247 (47.2)	0.11	27 (36.0)	225 (45.7)	0.12
TT	10 (13.2)	57 (10.9)	0.98	10 (13.3)	57 (11.6)	0.87
C	104 (68.4)	685 (65.5)	Ref.	103 (68.7)	645 (65.5)	Ref.
T	48 (31.6)	361 (34.5)	0.48	47 (31.3)	339 (34.5)	0.45

Data presented as *n *(%). Genotypic and allelic frequencies of the 1298 A>C and 677 C>T 5,10-methylene tetrahydrofolate reductase (*MTHFR*) gene polymorphisms in rheumatoid arthritis (RA) patients with (+) or without (-) cardiovascular events in the whole cohort and in patients with 5 years of follow up. Ref., reference.

**Table 3:** Frequencies of gene polymorphisms with or without cardiovascular events: 10 years follow up

	Cardiovascular event occurrence in RA patients					
	
	Overall			10 years follow up		
		
	+	-	*P *value	+	-	*P *value
*MTHFR *1298 A>C	(n = 77)	(n = 511)		(n = 64)	(n = 335)	
AA	29 (37.7)	257 (50.3)	Ref.	22 (34.4)	160 (47.8)	Ref.
AC	36 (46.8)	206 (40.3)	0.10	30 (46.9)	142 (42.4)	0.15
CC	12 (15.6)	48 (9.4)	0.03	12 (18.8)	33 (9.9)	0.01
A	94 (61.0)	720 (70.5)	Ref.	74 (57.8)	462 (69.0)	Ref.
C	60 (39.0)	302 (29.5)	0.02	54 (42.2)	208 (31.0)	0.01
*MTHFR *677 C>T	(n = 76)	(n = 523)		(n = 63)	(n = 347)	
CC	38 (50.0)	219 (41.9)	Ref.	31 (49.2)	152 (43.8)	Ref.
CT	28 (36.8)	247 (47.2)	0.11	24 (38.1)	153 (44.1)	0.37
TT	10 (13.2)	57 (10.9)	0.98	8 (12.7)	42 (12.1)	0.87
C	104 (68.4)	685 (65.5)	Ref.	86 (68.3)	457 (65.9)	Ref.
T	48 (31.6)	361 (34.5)	0.48	40 (31.7)	237 (34.1)	0.64

Data presented as *n *(%). Genotypic and allelic frequencies of the 1298 A>C and 677 C>T 5,10-methylene tetrahydrofolate reductase (*MTHFR*) gene polymorphisms in rheumatoid arthritis (RA) patients with (+) or without (-) cardiovascular events in the whole cohort and in patients with 10 years follow up. Ref., reference.

Both associations were independent of the time of follow up. Genotypic and allelic differences between RA patients with and without CV events found in the whole cohort were thus also observed in RA patients with 5 years follow up and 10 years follow up, respectively (Tables 2 and 3). In this regard, the *MTHFR *1298 C-allele frequency was increased in patients with CV events after 5 years (38.7% versus 30.3%; OR = 1.45; 95% CI = 1.00 to 2.10; *P *= 0.04) and 10 years (42.2% versus 31.0%; OR = 1.62; 95% CI = 1.08 to 2.43; *P *= 0.01) follow up, respectively.

As shown in Tables 2 and 3, however, no significant difference in the allelic and genotypic frequencies of the *MTHFR *677 C>T gene polymorphism was observed when RA patients were stratified according to the presence of CV events, regardless of follow-up duration.

### *MTHFR *gene polymorphisms and endothelial function

Endothelial function was assessed in 108 RA patients with no history of CV events at the time of the brachial ultrasonographic study. Patients were stratified according to the *MTHFR *genotypes. The mean value for the FMD percentage in this series of RA patients was lower than 7%. This result confirms the presence of endothelial dysfunction in long-standing RA patients from Northwest Spain [17,20].

**Figure 1:**
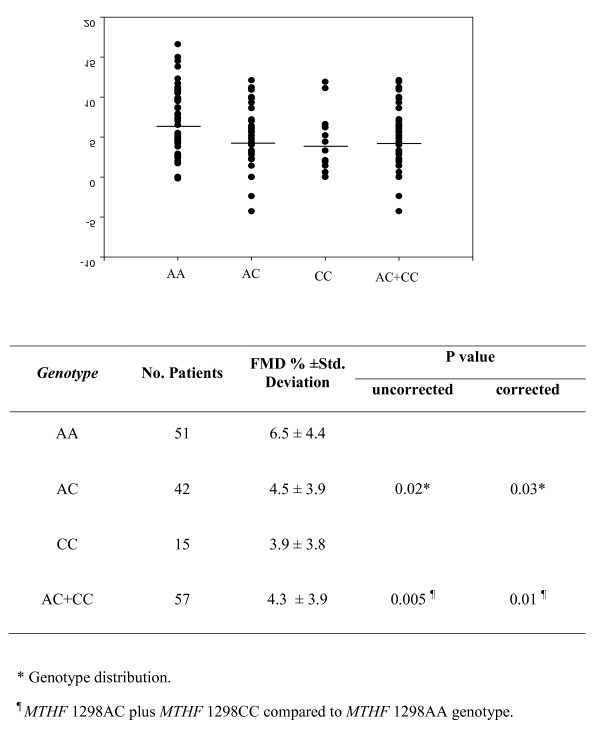
**Endothelium-dependent, flow-mediated (post-ischemia) vasodilatation in rheumatoid arthritis patients stratified according to genotype**. Study of endothelium-dependent, flow-mediated (post-ischemia) vasodilatation (FMD) in rheumatoid arthritis patients stratified according to the 5,10-methylene tetrahydrofolate reductase (MTHFR) 1298 genotypes. Individual and mean values (horizontal lines) expressed as the FMD percentage are shown.

With respect to the *MTHFR *677 C>T gene polymorphisms, RA patients homozygous for *MTHFR *677CC had lower values of FMD percentage (4.9 ± 4.2) than those carrying the *MTHFR *677CT genotype (FMD percentage = 5.9 ± 4.4) or the *MTHFR *677TT (FMD percentage = 3.6 ± 2.9), but the difference was not statistically significant (*P *= 0.22). When the *MTHFR *1298 A>C gene polymorphism was assessed, however, significant differences in FMD percentage values according to the different genotypes were observed (Figure 1). In this regard, RA patients carrying *MTHFR *1298 AC + *MTHFR *1298 CC (FMD percentage = 4.3 ± 3.9) had more severe endothelial dysfunction than those homozygous for the *MTHFR *1298 AA genotype (FMD percentage = 6.5 ± 4.4) (*P *= 0.005). Interestingly, this association was independent of the gender, age and duration of the disease, rheumatoid factor and traditional CV risk factors, since the result of the analysis of covariance test remains statistically significant (*P *= 0.01).

No significant differences were observed when the NTG percentage in RA patients was stratified according to the *MTHFR *677 C>T and the *MTHFR *1298 A>C gene polymorphism genotypes (data not shown).

## Discussion

Owing to the implication of homocysteine in the mechanisms associated with increased incidence of CV events in the general population, functional polymorphisms in the *MTHFR *gene have been proposed as potential candidates for atherosclerosis in RA, a disease associated with increased risk of CV events and CV mortality [1]. Interestingly, our results for first time show an implication for the *MTHFR *A1298C gene polymorphism in the increased risk of atherosclerosis of patients with RA. In this regard, patients homozygous for the *MTHFR *1298 CC genotype had increased risk of CV events at 5 and 10 years follow up when compared with those homozygous for the wild *MTHFR *1298AA genotype. Also, the presence of the mutant allele C of the *MTHFR *A1298C polymorphism was associated with increased risk of CV events. Moreover, patients carrying the *MTHFR *1298AC and *MTHFR *1298CC genotypes showed more severe endothelial dysfunction expressed by lower values of FMD percentage when compared with those homozygous for the *MTHFR *1298AA genotype. This finding is of particular importance since the presence of abnormal FMD percentage values is expression of endothelial dysfunction, which is a useful surrogate marker of subclinical atherosclerosis in patients with RA [20]. Interestingly, the *MTHFR *allele 1298C has also been found to be associated with a risk of early-onset coronary artery disease independent of homocysteine, folic acid, or vitamin B_12 _levels [22]. In this regard, Weisberg and colleagues reported that the *MTHFR *1298 mutation alone does not affect plasma homocysteine levels [23].

The molecular pathology of the missense A1298C mutation is not fully understood. This mutation results in an amino acid change of glutamate to alanine in the regulatory C-terminal domain of the enzyme, and is not manifested by hyperhomocysteinemia or associated with classic CV risk factors [12]. *In vitro*, *MTHFR *1298C carriers exhibited decreased enzyme activity - indicating the functional importance of the A1298C polymorphism, through a molecular mechanism that remains unknown [23]. Recent studies have disclosed that 5-methyl-tetrahydrofolate, the circulating metabolite of folic acid participating in homocysteine metabolism, rather than plasma or vascular homocysteine, seems to be a key regulator of endothelial nitric oxide synthase coupling and nitric oxide bioavailability in human vessels, suggesting that plasma homocysteine is an indirect marker of 5-methyl-tetrahydrofolate rather than a primary regulator of endothelial function [24]. These results question the direct role of circulating homocysteine as an atherosclerosis risk factor.

Unlike Rubini and colleagues, we did not observe an association of the *MTHFR *A1298C polymorphism with susceptibility to RA in the Spanish population [13]. The association of the *MTHFR *A1298C gene polymorphism with CV events in Spanish individuals with RA therefore seems to be independent of the potential role of this polymorphism in the susceptibility to the disease.

With regard to the *MTHFR *C677T gene variant, we did not find any significant association with susceptibility to RA. It was also the case when we assessed endothelial function or the presence of CV events. These findings do not support the previously reported association of this gene polymorphism with increased risk of premature coronary artery disease [25], endothelial dysfunction in patients undergoing coronary artery bypass graft surgery [24] and higher risk for stroke [26,27]. Nevertheless, it seems that this polymorphism is only associated with an increased risk of CV disorders under low-folate conditions, varying between different populations according to characteristic folate intake [28]. Although the present study included a reasonable sample size, however, the numbers decreased when patients with RA were stratified for CV events. This fact might explain the failure to see association of the C677T *MTHFR *gene polymorphism with CV events due to lack of power. Finally, replication of our findings in an independent dataset is needed to confirm the implication of the *MTHFR *A1298C gene polymorphism in the increased risk of atherosclerosis of patients with RA.

## Conclusions

The present study shows for the first time an association of *MTHFR *A1298C gene polymorphism with the risk of CV events and subclinical atherosclerosis manifested by the presence of endothelial dysfunction in patients with RA.

## Abbreviations

CI: confidence interval; CV: cardiovascular; FMD: endothelium-dependent, flow-mediated (post-ischemia) vasodilatation; MTHFR: 5,10-methylene tetrahydrofolate reductase; MTX: methotrexate; NTG: endothelium-independent (post-nitroglycerin) vasodilatation; OR: odds ratio; PCR: polymerase chain reaction; RA: rheumatoid arthritis; SNP: single nucleotide polymorphism; TNF: tumor necrosis factor.

## Competing interests

The authors declare that they have no competing interests.

## Authors' contributions

RP-M carried out genotyping, participated in the design of the study and in data analysis, and helped to draft the manuscript. CG-J performed the ultrasonographic studies, participated in the design of the study and in data analysis, and helped to draft the manuscript. TRV-R participated in the acquisition and interpretation of data. LR participated in the acquisition and interpretation of data. JAM-F participated in the acquisition and interpretation of data. BF-G has been involved in the acquisition and interpretation of data and in revising the data critically for important intellectual content. JL participated in the analysis of the data. JM and MAG-G made substantial contributions to conception and design of the study, acquisition of data and coordination, helped to draft the manuscript and gave final approval of the version to be published.
